# Tolvaptan in the Management of Acute Euvolemic Hyponatremia After Transsphenoidal Surgery: A Retrospective Single-Center Analysis

**DOI:** 10.3389/fendo.2021.689887

**Published:** 2021-05-24

**Authors:** Rita Indirli, Júlia Ferreira de Carvalho, Arianna Cremaschi, Beatrice Mantovani, Elisa Sala, Andreea Liliana Serban, Marco Locatelli, Giulio Bertani, Giulia Carosi, Giorgio Fiore, Leonardo Tariciotti, Maura Arosio, Giovanna Mantovani, Emanuele Ferrante

**Affiliations:** ^1^ Department of Clinical Sciences and Community Health, University of Milan, Milan, Italy; ^2^ Endocrinology Unit, Fondazione IRCCS Ca’ Granda Ospedale Maggiore Policlinico, Milan, Italy; ^3^ Department of Neurosurgery, Fondazione IRCCS Ca’ Granda Ospedale Maggiore Policlinico, Milan, Italy; ^4^ Department of Pathophysiology and Transplantation, University of Milan, Milan, Italy; ^5^ Department of Experimental Medicine, Sapienza University of Rome, Rome, Italy

**Keywords:** tolvaptan, syndrome of inappropriate antidiuresis, fluid restriction, transsphenoidal surgery, pituitary adenoma, hyponatremia

## Abstract

**Introduction:**

Syndrome of inappropriate antidiuresis (SIAD) can be a complication of hypothalamus-pituitary surgery. The use of tolvaptan in this setting is not well established, hence the primary aim of this study was to assess the sodium correction rates attained with tolvaptan compared with standard treatments (fluid restriction and/or hypertonic saline). Furthermore, we compared the length of hospital stay in the two treatment groups and investigated the occurrence of overcorrection and side effects including osmotic demyelination syndrome.

**Methods:**

We retrospectively reviewed 308 transsphenoidal surgical procedures performed between 2011 and 2019 at our hospital. We selected adult patients who developed post-operative SIAD and recorded sodium monitoring, treatment modalities and outcomes. Correction rates were adjusted based on pre-treatment sodium levels.

**Results:**

Twenty-nine patients (9.4%) developed post-operative SIAD. Tolvaptan was administered to 14 patients (median dose 15 mg). Standard treatments were employed in 14 subjects (fluid restriction n=11, hypertonic saline n=1, fluid restriction and hypertonic saline n=2). Tolvaptan yielded higher adjusted sodium correction rates (12.0 mmolL^-1^/24h and 13.4 mmolL^-1^/48h) than standard treatments (1.8 mmolL^-1^/24h, p<0.001, and 4.5 mmolL^-1^/48h, p=0.004, *vs.* tolvaptan). The correction rate exceeded 10 mmolL^-1^/24h or 18 mmolL^-1^/48h in 9/14 and 2/14 patients treated with tolvaptan, respectively, and in no patient who received standard treatments. No side effects including osmotic demyelination occurred. Tolvaptan was associated with a shorter hospital stay (11*vs.*15 days, p=0.01).

**Conclusions:**

Tolvaptan is more effective than fluid restriction (with or without hypertonic saline) and allows for a shortened hospital stay in patients with SIAD after transsphenoidal surgery. However, its dose and duration should be carefully tailored, and close monitoring is recommended to allow prompt detection of overcorrection.

## Introduction

Hyponatremia due to syndrome of inappropriate antidiuresis (SIAD) can be a complication of hypothalamus-pituitary surgery, with a variable incidence ranging from 1.8 to 37% in different series (1–5). Although most cases are mild and asymptomatic (4), a minority of patients can manifest headache, nausea, vomiting, irritability, confusion, concentration difficulties (6), and with moderate-severe forms stupor, seizures and coma, which require prompt correction of sodium levels (7). Consistently, hyponatremia is associated with longer hospital stay and increased cost of care (8).

Hyponatremia following pituitary surgery is generally treated with fluid restriction or, in severe or refractory cases, with 3% hypertonic saline, alone or in combination (5, 7, 9). Fluid restriction has limited efficacy, requires a long time to normalization of sodium levels, and is poorly tolerated (10, 11). Hypertonic saline is effective in rapidly increasing serum sodium concentration (10, 12, 13), but can lead to overly rapid correction (14). For this reason, 3% saline is not generally aimed at completely normalizing sodium levels, and it should be discontinued once serum sodium concentrations increase to a safe threshold or symptoms improve (11, 12, 15).

Vaptans are arginine-vasopressin receptor antagonists whose efficacy and safety has been demonstrated in randomized clinical trials for the treatment of hypervolemic and euvolemic hyponatremia (16, 17). The use of these medications in the post-operative setting (18, 19) and in critical neurological and neurosurgical adult patients (20, 21) has shown that vaptans allow correction of hyponatremia with the rare occurrence of adverse effects.

However, some peculiar aspects of SIAD following pituitary surgery need be taken into consideration before applying to this setting the results obtained when vaptans are used in other conditions. Firstly, SIAD is self-limiting in this setting and resolves within 2-5 days (22). In addition, hyponatremia can be isolated, follow or precede central diabetes insipidus in a biphasic or triphasic pattern, so that rapid changes in serum sodium and urine output can be observed in the post-operative period, and such changes may be worsened by treatments for either condition. For these reasons, close monitoring and prompt adjustment or discontinuation of ongoing treatments are recommended (5, 7).

Experience on the use of vaptans in this context is limited, and mostly based on case reports or small case series (23–25), while head-to-head trials comparing vaptans with standard treatments are lacking. In a retrospective analysis, the intravenous vasopressin receptor antagonist conivaptan and the oral antagonist tolvaptan provided a significantly higher rise in sodium levels compared to no treatment, whereas water restriction, salt tablets and 3% saline did not (2). Only one case of hypernatremia was reported with conivaptan. In a recently published observational study, Kleindienst et al. (26) compared tolvaptan at low (3.75 mg/die) and moderate doses (7.5 mg/die) with fluid restriction, showing tolvaptan to be more effective, but also associated with overly rapid correction in up to 44% patients. As no definitive conclusion can be drawn from current evidence, further studies on vaptans’ safety and efficacy are warranted.

In the present study, we retrospectively reviewed the management of hyponatremia developing after surgery of sellar lesions at the Neurosurgery Department of our hospital. The primary aim was to compare sodium correction rate between patients treated with tolvaptan and patients who received standard treatments (fluid restriction and 3% saline, alone or in combination). The secondary aim was to compare other parameters of safety and efficacy, i.e. length of hospitalization, overcorrection, occurrence of hypernatremia and side effects.

## Materials and Methods

### Study Design

We conducted a retrospective cohort study to compare outcomes associated with tolvaptan or standard treatment in the management of SIAD which develops after sellar surgery. To this purpose, we reviewed the clinical and biochemical data of surgical procedures performed between January 2011 and December 2019 at our tertiary pituitary center.

We selected adult patients who had no known disorders of sodium and water balance ahead of surgery and developed SIAD in the post-operative period. Any medications initiated in the post-operative period which could cause or contribute to hyponatremia (i.e. desmopressin in the 24 hours preceding hyponatremia onset, proton pump inhibitors, diuretics, infused hypotonic solutions…) (12, 15) were considered as exclusion criteria.

Data were extracted from clinical charts from the Neurosurgery Department and from follow-up outpatient visits. Age, sex, sellar lesion type and size, serum sodium monitoring during hospital stay, time of occurrence of hyponatremia, sodium nadir, treatment modalities, dose and time of administration, hyponatremia correction rates, treatments’ side effects, risk factors for osmotic demyelination, incidence of osmotic demyelination syndrome and length of hospitalization were recorded.

The study was conducted in accordance with the World Medical Association’s Declaration of Helsinki and approved by Milan Area 2 ethics committee (reference number 117612). Written informed consent was obtained from all individuals included in the study.

### Definitions

Postoperative hyponatremia was defined as sodium levels < 135 mmol/L occurring during the post-operative hospital stay. SIAD was defined according to current guidelines (12, 15, 27), i.e. hyponatremia occurring in a normovolemic patient with plasma osmolality <275 mOsmol/kg, urinary osmolality >100 mOsmol/kg with normal renal function, urinary sodium >30 mmol/L, after exclusion of uncorrected thyroid or adrenal insufficiency. SIAD could occur as an isolated disorder, or as the second phase of a biphasic or triphasic diabetes insipidus. The euvolemic state, the maintenance of stable vital signs (no increase in heart rate, reduction in blood pressure or orthostatic hypotension) and a low-normal urine output were considered for the differential diagnosis with cerebral salt-wasting syndrome (12, 15).

Hyponatremia was classified as “mild” for serum sodium concentrations between 130 and 135 mmol/L, “moderate” between 125 and 129 mmol/L, and “profound” below 125 mmol/L. Moderate symptoms included nausea without vomiting, confusion, headache. Severe symptoms included vomiting, cardiorespiratory distress, somnolence, seizures and coma (12).

Fluid restriction was defined as fluid intake less than 1000 mL in 24 hours, including intravenous fluids.

Hypertonic saline was administered as a continuous venous infusion. Correction rate was established according to Adrogué-Madias formula (28) and infusion was discontinued when symptoms improved, serum sodium concentration increased by a total of 10 mmol/L or reached 130 mmol/L (12).

Treatment failure was arbitrarily defined as a further reduction of sodium levels in the 24 hours following treatment administration.

Side effects of treatments were screened, including nausea, thirst, dry mouth, orthostatic hypotension, dizziness, hyperkalemia, raised liver or renal function tests (16, 27). Overcorrection was defined as a sodium correction rate >10 mmolL^-1^/24h or >18 mmolL^-1^/48h (10, 12). Hypernatremia was defined as sodium levels >145 mmol/L. We considered the following as risk factors for osmotic demyelination: serum sodium ≤105 mmol/L; hypokalemia; alcoholism; malnutrition; advanced liver disease (10). Osmotic demyelination syndrome was diagnosed with typical neurological features manifesting after the correction of sodium levels (29), and with focal cerebral demyelination diagnosed with magnetic resonance imaging.

### Surveillance and Procedures

Per institution protocol, all patients underwent a thorough evaluation of pituitary function preoperatively, including baseline pituitary hormones assessment, a corticotropin stimulation test, evaluation of serum electrolytes and daily fluid balance. Patients in whom adrenal or thyroid insufficiency was diagnosed, glucocorticoid replacement therapy or levothyroxine were initiated.

All surgical procedures were performed by 2 experienced neurosurgeons (M.L., G.B.) using the endonasal endoscopic transsphenoidal approach. Stress dose hydrocortisone was administered intravenously at induction of general anesthesia, and glucocorticoid replacement therapy was continued in all patients in the post-operative period, irrespective of pre-operative hypothalamus-pituitary-adrenal axis assessments. In addition, thyroxine levels were reassessed during hospital stay in patients with macroadenoma or other large sellar lesions and in those who underwent extensive surgical manipulation of the hypothalamus-pituitary structures, and levothyroxine was administered when central hypothyroidism developed. Therefore, preemptive glucocorticoid replacement therapy and prompt thyroid hormone replacement as needed, were guaranteed to all patients, excluding uncorrected adrenal or thyroid insufficiency as potential causes of hyponatremia. Pituitary function was reassessed comprehensively 8 to 12 weeks after surgery, and therapies were adjusted accordingly.

During the hospital stay, serum sodium levels and urine output were monitored daily, every 12 hours if diabetes insipidus or hyponatremia occurred, or every 6 hours if 3% saline or tolvaptan therapy was initiated.

When hyponatremia developed, fluid restriction, 3% saline (alone or in combination), or tolvaptan were prescribed, following an endocrinologist consultation (G.M., E.F.).

Tolvaptan was administered at the dose of 15 mg or 7.5 mg once a day. Before 2016 the higher dose was most frequently used, while after 2016 the 7.5 mg dose was the most prescribed one, based on the progressively acquired experience on the use of lower dosages. No other therapy was administered in combination with tolvaptan, and any prior treatment (first line fluid restriction and/or 3% saline) was discontinued when tolvaptan was prescribed as second line medication. Patients on tolvaptan therapy were allowed free access to water and were instructed to drink to satisfy thirst.

After normalization of sodium levels, hospital discharge was delayed by 2 days in all patients to maintain monitoring of fluid balance and serum electrolytes.

### Outcomes Measures

For our primary outcome, we calculated and compared the median 24-hour and 48-hour correction rates, i.e. the change in serum sodium concentrations from baseline (before treatment initiation) to 24 or 48 hours after treatments’ administration.

For secondary outcomes, we compared: i) length of hospitalization, i.e. the time from surgical procedure to hospital discharge; ii) safety parameters, including sodium overcorrection, hypernatremia, osmotic demyelination syndrome and treatment side effects.

### Sample Size

In the study by Kleindienst et al. on SIAD management following pituitary surgery (26), the median sodium increment per 24 hours was 3.5 mmol/L (range 1-12) in patients treated with fluid restriction, and 7.8 mmol/L (range 2-14) in patients receiving 7.5 mg tolvaptan. Based on these data, we calculated a sample size of 11 subjects per group (tolvaptan *vs.* standard treatments) when setting a power of 90% and a 5% probability of α error.

### Statistics

Quantitative variables were expressed as median (min-max range). Absolute (percentage) frequencies were reported for categorical variables. The distribution of continuous variables was analyzed by Shapiro-Wilk and D’Agostino-Pearson tests. Quantitative variables were compared by paired on unpaired t-test (2 groups) or analysis of variance (ANOVA, 3 groups) if normally distributed; Mann-Whitney test, Wilcoxon matched-pairs signed rank test (2 groups), or Kruskal-Wallis test (3 groups) were employed for skewed distributions. Two-way repeated measures ANOVA was used to determine how response (i.e. sodium variation) was affected by two factors (time, treatment), and analysis of covariance (ANCOVA) to adjust for covariate (pre-treatment serum sodium). Association was assessed by logistic regression analysis. Frequencies of qualitative variables were compared by Fisher’s exact test in 2x2 contingency tables, or by Chi square among three groups. A 2-sided p-value was considered statistically significant when less than 0.05.

Analysis was performed with GraphPad Prism (version 9).

## Results

**Figure 1:**
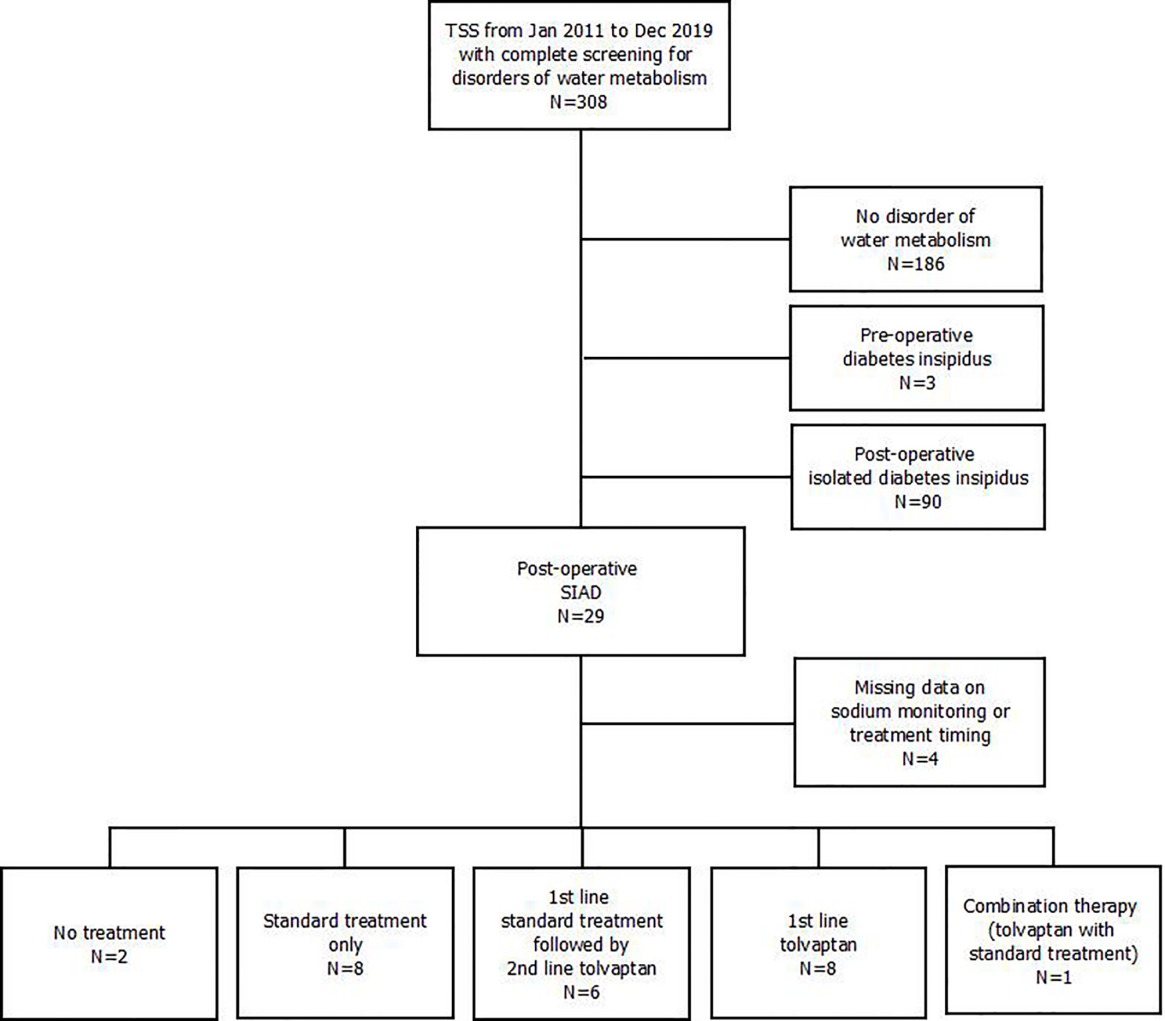
Flow-chart outlining participants’ selection. TSS, transsphenoidal sellar surgery; SIAD, syndrome of inappropriate antidiuresis.

Data on pre- and post-operative disorders of water metabolism were available in 308 patients who underwent TSS between January 2011 and December 2019 (**Figure 1**).

Twenty-nine patients (9.4%) developed post-operative SIAD (median urinary sodium 133 mmol/L [range 52-241], urine osmolality 528 mOsm/Kg [397-660], serum osmolality 267 mOsm/Kg [259-275], urine output 1500 mL per day [600-2000]).

For 4 patients information on hyponatremia management was incomplete, so they were excluded from analysis. Of the remaining patients, hyponatremia resolved spontaneously without any specific treatment in 2: they both presented mild hyponatremia after resection of non-functioning pituitary macroadenomas, with spontaneous resolution within 24 hours.

Eight patients received standard treatment only (group A; fluid restriction only, N=6; fluid restriction and hypertonic saline, N=2), 6 patients were administered tolvaptan as second line after failure of standard treatment (group B; fluid restriction N=5 and 3% saline N=1), whereas 8 were treated with tolvaptan as first line therapy (group C). In group B, patients were switched to tolvaptan 24 hours after receiving standard treatments. In patients receiving hypertonic saline, the reason for stopping infusion was the finding of serum sodium ≥ 130 mmol/L (with a total sodium increase of 3, 4 and 7 mmol/L in the three patients respectively); thereafter, fluid restriction alone (2 patients in group A) or tolvaptan (1 patient in group B) were employed.

Finally, another patient was initially treated with tolvaptan 15 mg for profound hyponatremia (112 mEq/L). After 12 hours, despite a sodium increase to 120 mEq/L, the patient presented generalized tonic-clonic seizures which required addition of 3% hypertonic saline infusion, with subsequent sodium normalization and symptom relief. Given that this was the only case of combined treatment (tolvaptan and hypertonic saline), this patient was excluded from further analysis.

Patients included in the analysis did not develop any other post-operative complication besides disorders of sodium and water balance.

### Hyponatremia Management

Baseline characteristics of the three groups (A, B, C) are summarized in **Table 1**.

**Table 1:** Demographics and baseline characteristics of patients treated with standard treatment only (A, N=8), first line standard treatment + second line tolvaptan (B, N=6), and first line tolvaptan (C, N=8).

	A, Standard treatment N=8	B, First line standard treatment + second line tolvaptan N=6	C, First line tolvaptan N=8	p-value
Age, *years*	67 (37-74)	57 (43-66)	59 (43-77)	0.69
Female, *N*	4	5	3	0.22
Pituitary adenoma, *N*	8	6	7	0.40
Nonfunctioning, *N* GH-secreting, *N* PRL-secreting, *N* ACTH-secreting, *N*	5 1 1 1	4 2 0 0	4 2 0 1	0.78
Macroadenoma, *N*	7	6	6	0.64
Craniopharyngioma, *N*	0	0	1	0.40
Pre-operative pituitary insufficiency, *N*	4	1	2	0.36
P.o. pituitary insufficiency, *N*	6	1	6	**0.05^#^**
P.o. diabetes insipidus, *N*	2	4	4	0.29
Triphasic, *N*	1	1	1	0.79
Permanent, *N*	1	0	1	0.33
Hyponatremia onset time, *p.o. days*	5 (3-7)	6 (4-7)	6 (4-7)	0.87
Time to treatment administration, *p.o. days*	7 (3-10)	6 (5-8)	6 (6-8)	0.87
Lag time, *days*	0 (0-5)	0 (0-2)	0 (0-2)	0.92
S-Na at hyponatremia onset, *mmol/L*	131 (126-134)	128 (127-134)	128 (119-134)	0.28
Pre-treatment S-Na, *mmol/L*	131 (125-134)	129 (127-130)	125 (112-129)	**0.004^**
S-Na nadir, *mmol/L*	130 (120-134)	125 (122-129)	125 (112-129)	0.05*
Time to S-Na nadir, *p.o. days*	6 (3-7)	7 (6-9)	6 (6-8)	0.30
Biochemical severity at treatment administration, *N*				
Mild	6	2	0	
Moderate	2	4	5	**0.01^§^**
Profound	0	0	3	
Symptom severity, *N*				
Asymptomatic	6	5	5	
Moderate Severe	2 0	0 1	1 2	0.10
At risk for myelinosis, *N*	0	0	0	–

^#^A vs. B p=0.10; B vs. C p=0.10; A vs. C p>0.99. ^^^A vs. B p=0.75; **B vs. C p=0.03; A vs. C p=0.004**. ^§^A vs. B p=0.28; B vs. C p=0.09; **A vs. C p=0.01**. *A vs. B p=0.33; B vs. C p=0.66; **A vs. C p=0.045**. Median (and range, in brackets) are reported for quantitative variable; absolute frequency is reported for categorical variables. P-values equal to or below 0.05 are reported in bold characters. GH, growth hormone; PRL, prolactin; ACTH, adrenal corticotropic hormone. Pituitary insufficiency, any pituitary hormone deficiency (growth hormone deficiency, secondary adrenal insufficiency, central hypothyroidism, hypogonadotropic hypogonadism), whether isolated or multiple. p.o., post-operative. Lag time, time from hyponatremia onset to treatment administration. S-Na, serum sodium. For group B, when “pre-treatment” or “treatment administration” are indicated, the values relating to first line standard treatments are reported.

At hyponatremia onset, median serum sodium was in the range of mild hyponatremia in group A, and of moderate hyponatremia in groups B and C, however, this difference was not statistically significant (**Table 1**).

Treatments were started within 48 hours of hyponatremia onset, except for one patient in group A (**Table 1**). At the time of treatment administration, group C patients showed a further decrease of sodium levels [125 (112–129) mmol/L] compared to hyponatremia onset (p=0.05), unlike group A and B patients (p=0.18 and p=0.50 *vs.* hyponatremia onset, respectively). Tolvaptan was chosen as the first-line treatment in group C, in which profound hyponatremia and severe symptoms were observed more frequently.

**Figure 2:**
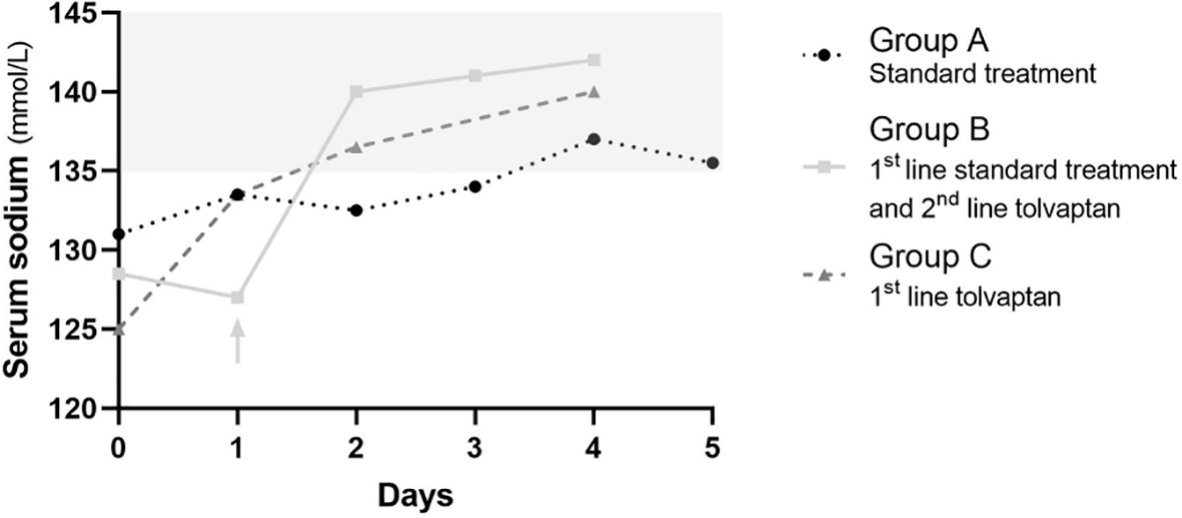
Serum sodium changes overtime in patients receiving standard treatment only (A, black dotted line with circles), first line standard treatment and second line tolvaptan (B, continuous light grey line with squares), and first line tolvaptan (C, dashed dark grey line with triangles). Median values are reported. Day 0 is the day the first line treatment was started. The light grey arrow indicates the switch from standard treatment to tolvaptan in group (B). The light grey area outlines the normal range of serum sodium concentration.

Sodium nadir occurred before initiation of standard treatment in group A and before initiation of tolvaptan in group C, but after administration of standard treatment in group B, and these patients were then switched to second line tolvaptan (**Figure 2**).

### Correction Rates

In the following analysis, group B patients were included in both the standard treatment group (extrapolating data on the first line standard treatments; STD, N=14), and the tolvaptan group (extrapolating data on the second line tolvaptan therapy; TLV, N=14). Then, sodium changes occurring after standard treatments and after tolvaptan administration were compared (**Table 2**).

**Table 2:** Outcomes of standard treatments (STD, N=14, including fluid restriction and/or hypertonic saline), tolvaptan (TLV, N=14), and fluid restriction only (excluding hypertonic saline, N=11) in the management of post-operative hyponatremia.

	STD (N=14)	TLV (N=14)	p-value (STD vs. TLV)	Fluid restriction (N=11)	p-value (Fluid restriction *vs.* TLV)
Timing of administration, *p.o. days*	6 (3 - 10)	7 (6 - 9)	0.24	7 (3 – 10)	0.42
Pre-treatment S-Na, *mmol/L*	130 (125-134)	125 (112-131)	**0.003**	130 (128 - 134)	**<0.001**
S-Na at 24-h, *mmol/L*	129 (124-140)	137 (129-151)	**0.02**	129 (124 – 140)	**0.02**
24-h correction rate, *mmolL^-1^/24h*	+1 (-5 to +8)	+12.5 (+5 to +26)	**<0.001**	-1 (-5 to +8)	**<0.001**
Adj. 24-h correction rate, *mmolL^-1^/24h*	+1.8	+12.0	**<0.001**	+0.8	**<0.001**
24-h overcorrection (>10 mmolL^-1^/24h), *N (%)*	0 (0)	9 (64)	**<0.001**	0 (0)	**0.001**
S-Na normalized at 24-h, *N (%)*	3 (21)	7 (50)	0.12	3 (27)	0.24
Hypernatremia at 24-h, *N (%)*	0 (0)	1 (7)	>0.99	0 (0)	>0.99
S-Na at 48-h, *mmol/L*	131 (122-140)	138 (132-144)	**0.02**	132 (122 – 140)	0.06
48-h correction rate, *mmolL^-1^/48h*	+4.5 (-8 to +9)	+15 (+7 to +20)	**<0.001**	+2 (-8 to +9)	**<0.001**
Adj. 48-h correction rate, *mmolL^-1^/48h*	+4.5	+13.4	**0.004**	+3.9	**0.01**
48-h overcorrection (>18 mmolL^-1^/48h), *N (%)*	0/8 (0)	2/14 (14)	0.52	0/6 (0)	>0.99
S-Na normalized at 48-h, *N (%)*	2/8 (25)	12/14 (86)	**0.008**	2/6 (33)	**0.04**
Hypernatremia at 48-h, *N (%)*	0 (0)	0 (0)	–	0 (0)	–
Side effects, *N (%)*	0 (0)	0 (0)	–	0 (0)	–
Osmotic demyelination, *N (%)*	0 (0)	0 (0)	–	0 (0)	–

p.o., post-operative. Adj., correction rates adjusted by pre-treatment sodium levels. S-Na, serum sodium. 24-h, 24 hours after initiation of treatment. 48-h, 48 hours after initiation of treatment. Median (and range, in brackets) is reported for quantitative variable; absolute frequency (and percentage, in brackets) is reported for categorical variables. P-values equal to or below 0.05 are reported in bold characters. Follow-up at 48 hours from treatment initiation was available in 8 patients of the STD group, as 6 were switched to TLV.

**Figure 3:**
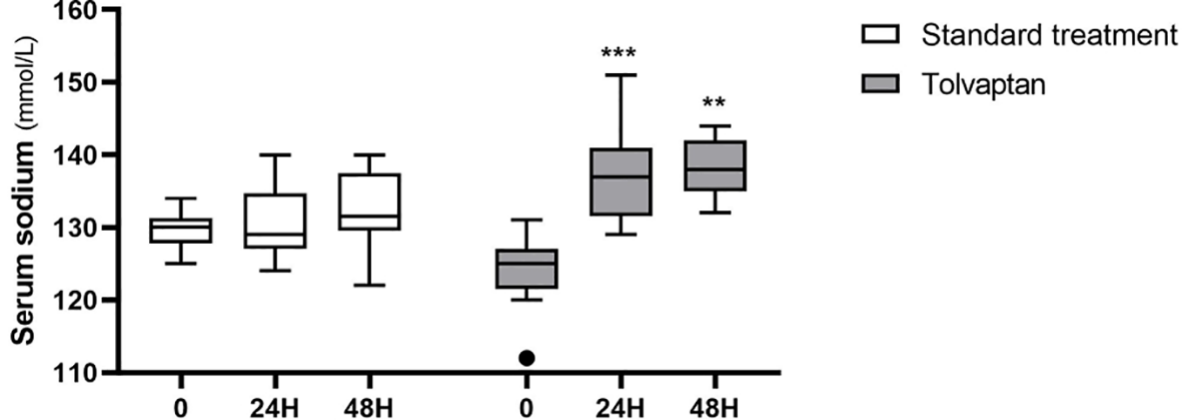
Serum sodium 24 hours and 48 hours after administration of standard treatments (white boxes) or tolvaptan (grey boxes). **p < 0.01; ***p < 0.001, *vs.* pre-treatment sodium levels (time 0).

In the first 24 hours, median sodium levels changed from 130 (125-134) mmol/L to 129 (124-140) mmol/L in the STD group (p=0.32), and from 125 (112-131) mmol/L to 137 (129-151) mmol/L in the TLV group (p<0.001; two-way repeated measures ANOVA: p<0.001 for time; p<0.001 for group-time interaction) (**Figure 3**). The median correction rate was significantly higher in the TLV group (+12.5 [+5 to +26] mmolL^-1^/24h) compared to the STD group (+1.0 [-5 to +8] mmolL^-1^/24h, p<0.001). As pre-treatment sodium levels were significantly different between treatment groups, and correction rates are inversely associated with baseline sodium levels (14, 30), we performed ANCOVA analysis including pre-treatment sodium level as a covariate. The mean adjusted correction rate was +1.8 (95% confidence interval, CI, -1.2 to +4.8) mmolL^-1^/24h for STD and +12.0 (95% CI +9.0 to +15.0) mmolL^-1^/24h for TLV (p<0.001).

Follow-up at 48 hours from treatment initiation was available in 8 patients of the STD group, as the remaining patients were switched to TLV.

After 48 hours, median sodium levels rose to 131 (122-140) mmol/L in patients receiving STD treatment (p=0.21 *vs.* baseline) and to 138 (132-144) mmol/L in subjects treated with TLV (p=0.001 *vs.* baseline; two-way repeated measures ANOVA: p<0.001 for time; p<0.001 for group-time interaction) (**Figure 3**). The median correction rate was significantly higher in the TLV group (+15 [+7 to +20] mmolL^-1^/48h) compared to the STD group (+4.5 [-8 to +9] mmolL^-1^/48h, p<0.001). The mean correction rates adjusted by pre-treatment sodium levels were +4.5 (95% CI +0.6 to +8.5) mmolL^-1^/48h for STD and +13.4 (95% CI +10.1 to +16.6) mmolL^-1^/48h for TLV (p=0.004).

After excluding patients treated with hypertonic saline, the mean adjusted correction rates with fluid restriction (N=11) were +0.8 mmolL^-1^/24h (95% CI -3.0 to +4.5, p<0.001 *vs.* TLV) and +3.9 mmolL^-1^/48h (95% CI -1.4 to +9.1, p=0.01 *vs.* TLV).

### Secondary Outcomes

The 24-hour correction rate exceeded 10 mmolL^-1^/24h in 9 out of 14 patients in the TLV group and in none of the STD group (p<0.001, **Table 2**). In a multinomial logistic regression, the treatment group was significantly associated with the risk of 24-hour overcorrection independently of pre-treatment sodium levels (p=0.02).

The 48-hour correction rate exceeded 18 mmolL^-1^/48h in 2 patients receiving TLV. One patient who received tolvaptan developed hypernatremia within 24 hours. No side effect, osmotic demyelination syndrome or signs of osmotic myelinosis on post-operative magnetic resonance imaging were recorded in either treatment arm.

After 48 hours, sodium levels were normalized in 12 of the 14 patients in the TLV group and in 2 of the 8 patients in the STD group (p=0.008).

After excluding patients receiving hypertonic saline, these outcomes were confirmed when comparing TLV with fluid restriction (**Table 2**).

The mean length of hospital stay was 11 days (SD 3) in patients receiving tolvaptan, 15 days (SD 3) in patients receiving standard treatment only (i.e. 3% saline and/or fluid restriction) (p=0.01 *vs.* tolvaptan) and 14 days (SD 4) in patients managed with fluid restriction only (p=0.07 *vs.* tolvaptan).

### Tolvaptan Dosage

Eleven patients received one single dose of tolvaptan (7.5 mg N=7, and 15 mg N=4). Three patients received 2 doses 24 hours apart: both 7.5 mg, N=1; both 15 mg, N=1; first dose 15 mg and second dose 7.5 mg, N=1.

#### Tolvaptan 7.5 mg *vs.* 15 mg

Pre-treatment sodium levels were comparable between patients receiving first dose tolvaptan 7.5 mg (126 [112-131] mmol/L, N=8) and 15 mg (125 [122-129] mmol/L, N=6; p=0.81), as was the median correction rate (+12.5 [+5 to +17] mmolL^-1^/24h, and +12 [+7 to +26] mmolL^-1^/24h, respectively; p=0.91). Overcorrection was observed in 6 of the 8 subjects receiving the lower dose, and in 3 of the 6 subjects taking the higher dose (p=0.58). Twenty-four hours following the first administration, sodium was normalized in 5 patients treated with 7.5 mg and in 3 patients treated with 15 mg tolvaptan (p>0.99). One patient who received 15 mg developed hypernatremia.

#### One Dose *vs.* Two Doses

Pre-treatment sodium levels were comparable between patients receiving one single dose (125 [112-131] mmol/L) and patients receiving 2 doses (125 [122-126] mmol/L, p=0.73). Two patients in the 1-dose group, but no patient in the 2-dose group, presented triphasic diabetes insipidus.

In patients receiving one dose, sodium levels rose to 139 [129-151] mmol/L in the first 24 hours, with sodium normalized in 7/11 patients, overcorrection in 9, and hypernatremia in 1. In these patients tolvaptan was discontinued. Among patients receiving two tolvaptan doses, sodium increased to 130 (130-133) mmol/L in the first 24 hours (p=0.05 *vs*. 1-dose group); no patient displayed sodium normalization, overcorrection or hypernatremia, and a second dose of tolvaptan was provided. At 48 hours from first administration, sodium reached the level of 138 (132-142) mmol/L in the 1-dose group, and 140 (138-142) mmol/L in the 2-dose group (p=0.35), with comparable correction rates (+14 [+6 to +20] and +14 [+13 to +20] mmolL^-1^/48h respectively, p=0.59). Sodium was normalized in 9/11 patients and in 3/3 patients in the respective groups. One patient in each group displayed overcorrection over 48 hours (p=0.40), but no patient developed hypernatremia at this timepoint. Two patients treated with one dose (7.5 mg) presented hyponatremia recurrence after initial normalization.

## Discussion

In the present study, we compared tolvaptan with standard treatments (fluid restriction and hypertonic saline) in the management of SIAD complicating the post-operative course of sellar surgery. Our results show that tolvaptan yielded higher sodium correction rates and allowed a shorter hospital stay in the absence of side effects, despite a higher incidence of overcorrection.

The incidence of SIAD after transsphenoidal procedures varies from 1.8% to 37% in different series (1–5), and fluid restriction is the most widely used monotherapy treatment in hyponatremia, but also the least effective (31, 32). Slow rates of sodium correction have been associated with increased mortality (33), and effective treatment strategies are recommended to prevent worsening of hyponatremia (34) and the prolongation of hospital stay.

In this study, tolvaptan produced a rapid correction of serum sodium in patients presenting with moderate-profound hyponatremia, and in refractory cases. Although standard treatments (mainly fluid restriction) provided slow normalization of serum sodium in the majority of patients with a mild biochemical presentation, they were ineffective in most subjects with moderate hyponatremia. Tolvaptan performed better than fluid restriction (with or without hypertonic saline) in all the efficacy outcomes considered, as it provided significantly higher 24-hour and 48-hour sodium correction rates, which were confirmed after adjustment by pre-treatment sodium levels, and was associated with a reduction in length of hospital stay. These results are in line with previously published research (26, 35). In the study by Jahangiri et al. (2), only vaptans produced a significant rise in sodium levels compared to no treatment, while standard measures did not. Barber et al. (36) reported that hypertonic saline was not significantly associated with a shortened time to resolution of hyponatremia, while fluid restriction predicted prolonged hyponatremia duration. Consistently, in some patients in our series fluid restriction resulted in no change or even in a decrease of sodium levels during the first 24 hours of therapy. In the study by Zada et al. (4), fluid restriction alone was effective in asymptomatic outpatients with moderate-profound hyponatremia after transsphenoidal surgery. However, this is only apparently opposite to our results, as in the same cohort, the addition of hypertonic saline was needed in symptomatic patients who were readmitted to the hospital after failure of fluid restriction monotherapy.

Interestingly, in our study, the adjusted correction rate for tolvaptan was 12.0 mmolL^-1^/24h, which is higher than previously reported (31, 32). Indeed, Kleindienst et al. documented that tolvaptan 7.5 mg increased sodium by 7.8 mmol/L/24h. However, only a minority of patients was affected by profound hyponatremia in that cohort, as opposed to ours, and baseline serum sodium is known to be inversely associated with the rapidity of correction (30, 37).

Current guidelines advocate for a correction threshold of 10 mmol/L in the first 24 hours and of 18 mmol/L in the first 48 hours (10, 12, 15). Since in our cohort tolvaptan led to overcorrection in 64% of patients, potential concerns for safety may arise, especially when tolvaptan was administered later than 48 hours from hyponatremia onset. Indeed, cerebral demyelination can ensue with overcorrection of severe hyponatremia of sufficient duration to allow brain volume adaptation, usually ≥48 hours (28, 29, 38, 39). Conversely, the relowering of serum sodium is not generally indicated for overcorrection of acute hyponatremia (10), as the risks of brain edema and osmotic demyelination should be carefully weighed in this setting (12, 15). Following transsphenoidal surgery, patients can develop acute hyponatremia and reach sodium levels at high risk for osmotic demyelination only in a minority of cases (36), thus the risk of this complication is generally low (12, 40). In our series, no patient was at high risk for nor developed osmotic demyelination syndrome.

According to our results we could suggest that, whenever tolvaptan is chosen for the management of SIAD after pituitary surgery, close monitoring is needed and strategies to employ tolvaptan safely should be implemented. In this perspective, administering tolvaptan within 48 hours from hyponatremia onset may minimize the risks carried by the possible occurrence of overly rapid correction. Therefore, in patients who develop profound hyponatremia acutely, tolvaptan could be considered as first-line treatment, as preventing a further drop in sodium levels and pursuing an effective correction of hyponatremia are paramount in this setting. On the other hand, in patients without profound hyponatremia, standard therapies may be attempted, but again close monitoring is advocated and switch to tolvaptan should not be delayed if first-line treatments prove ineffective. However, the choice of treatment is further complicated by the regulatory policy for tolvaptan prescription, which may variably limit its use in different states. In the authors’ country, for instance, tolvaptan can only be prescribed in moderate-profound, but not mild hyponatremia (41). Larger studies are warranted to better define the clinical and biochemical features which should guide treatment selection.

Since adequate titration of tolvaptan is crucial (42), we investigated whether different tolvaptan doses could impact outcomes. With the limitation of small sample size, we observed that 7.5 mg and 15 mg tolvaptan provided comparable results, but only one patient receiving 15 mg developed hypernatremia. Based on these preliminary results, we may suggest that the lower dose may be adequate for the management of SIAD following pituitary surgery. Likewise, Kleindienst et al. (26) reported that tolvaptan 3.75 mg and 7.5 mg daily had similar rates of success, but a higher risk of overcorrection was documented in the group receiving 7.5 mg, so that even the lowest dose may be safely and effectively employed. Finally, despite comparable pre-treatment sodium levels, patients’ response to the first dose of tolvaptan was heterogeneous and unpredictable. Some patients developed overcorrection or hypernatremia and required therapy discontinuation, while other patients needed a second dose to achieve normal sodium levels; moreover, hyponatremia relapsed after initial sodium normalization in two cases. Triphasic diabetes insipidus, with a polyuric phase following SIAD, may contribute to this variability, as it may add to the pharmacological effect of tolvaptan by enhancing free water clearance and raising serum sodium in some patients. Moreover, in this setting, hyponatremia *per se* is self-limiting and its severity and duration may vary, probably in relation to the extent of hypothalamus, stalk and neurohypophysis manipulation and damage. As we did not identify any factor which could predict the subsequent course of hyponatremia, we recommend that any treatment to correct hyponatremia be performed with particular caution, and a close monitoring and a tailored management be guaranteed to all patients.

The major limitations of this study result from its retrospective design. First, biochemical severity was not comparable between patients receiving standard treatments and tolvaptan. This reflects our institutions’ practice, as treatment selection is partially guided by the severity of hyponatremia. Nonetheless, a standardized protocol is lacking and this constitutes one more limitation. However, it should be noted that the treatment decision was up to 2 experienced physicians only, and clear-cut indications on the use of vaptans in this setting have not yet been released. Due to the relatively small sample size, we could not conduct a propensity score analysis to account for baseline treatment groups’ heterogeneity. Nevertheless, pre-treatment heterogeneity was instrumental in showing that the biochemical severity should be considered in the choice of treatment. Additionally, we adjusted sodium correction rates by baseline sodium levels, and confirmed significant differences between treatment groups. Finally, only a minority of patients received 3% hypertonic saline; in these patients, treatment was aimed at reaching safe, but not normalized sodium levels, as recommended (12), which made the addition of other treatments always necessary after discontinuation of 3% saline. This outlines a bias of the study and, for all these reasons, no conclusion on the comparison between 3% saline administration and tolvaptan can be drawn.

In conclusion, hyponatremia is a significant complication of hypothalamus-pituitary surgery, as it impacts on patients’ clinical course, length of hospital stay and costs. For all these reasons, a prompt and effective correction of acute hyponatremia is warranted, but clear indications on the best management strategy are lacking. In this study we confirmed that tolvaptan may be more effective than fluid restriction (with or without hypertonic saline), in ensuring normalization of sodium levels and a shorter hospital stay. Sodium overcorrection may be a concern following tolvaptan administration, but side effects, including osmotic demyelination, are rare. We suggest that early administration of tolvaptan at the lowest effective dose may be considered, but close monitoring is recommended. Prospective interventional studies are needed to define which patients are the best candidate for treatment with tolvaptan, the adequate dose, efficacy and safety in this specific setting.

## Data Availability Statement

The raw data supporting the conclusions of this article will be made available by the authors, without undue reservation.

## Ethics Statement

The studies involving human participants were reviewed and approved by Milan Area 2 ethics committee. The patients/participants provided their written informed consent to participate in this study.

## Author Contributions

ML, GB, EF, GM, and ES performed diagnosis, surgical and medical treatment, and follow-up. RI, JF, BM, and EF collected and analyzed clinical data, and prepared the manuscript. MA, GM, EF, AC, AS, GC, GF, and LT critically revised the manuscript. All authors contributed to the article and approved the submitted version.

## Funding

This work was supported by Ricerca Corrente Funds from the Italian Ministry of Health.

## Conflict of Interest

The authors declare that the research was conducted in the absence of any commercial or financial relationships that could be construed as a potential conflict of interest.

The reviewer ES declared a shared affiliation with one of the authors, GC, to the Editor at time of review.
